# Minimally altering a critical kinase for low-phytate maize

**DOI:** 10.1038/s41598-020-63016-5

**Published:** 2020-04-14

**Authors:** Alla Singh, Chikkappa Karjagi, Sujay Rakshit

**Affiliations:** ICAR-Indian Institute of Maize Research, P.A.U. Campus, Ludhiana, 141004 India

**Keywords:** Computational biology and bioinformatics, Computational models

## Abstract

Nutritional security is of vital importance for combating malnutrition and catering to increasing energy demands. Phytic acid is considered an anti-nutrient, which sequesters important metal ions, limiting their bioavailability. The *lpa* mutants of maize contain reduced phytate, thus increase its nutritive value. But low phytate is accompanied by negative pleiotropic effects. This article discusses the importance of *lpa2* gene amongst available options, for precise DNA editing to simultaneously improve nutrition and avoid pleiotropic effects.

## Introduction

One major objective of millennium development goals (MDGs) is the eradication of extreme poverty and hunger. A call was made in the form of sustainable development goals (SDGs) to achieve zero hunger by 2030. Consistent and sustained efforts are being made across the globe to achieve food security, mainly in developing countries of Asia, Africa and Latin America. More effort is needed if the SDGs are to be met timely^1^. Maize is an important staple crop as food and feed in many parts of the world. In developing countries, malnutrition is a public health problem. The rapidly expanding population on one hand, and the capacity of middle income groups to pay for higher nutrition on the other, demands production of high-quality foods. Thus of late, more emphasis is being given on the quality of food for humans and feed for animals. Improvement of nutritional value of food crops, either by enhancing the nutrient levels through bio-fortification or by reducing the anti-nutritional factors like phytates would substantially increase their biological value, either due to improved digestibility and/or bioavailability^2,3^. In case of maize, phytic acid is one such anti-nutritional factor which sequesters important metal ions like Mg^++^, Fe^++^, Zn^++^, K^+^ etc., thus reducing the bioavailability of these micronutrients^4^.

Phytic acid is the hexa-phosphorylated form of *myo*-inositol. Chemically, phytic acid is the *myo*-inositol (1,2,3,4,5,6)-hexakisphosphoric acid. The major portion of phytic acid is stored in seeds, mainly in the form of mixed salts of mineral cations, referred to as phytate. In maize, more than 80% of phytic acid is stored in seed germ, it acts as the reservoir of phosphorous and inositol^5^. Phytic acid is synthesized by two pathways, namely lipid- dependent (operates in all plant organs) and lipid- independent (predominant in seeds)^6^. Phytic acid is packed into storage vacoules and other organelles. Due to sequestration of metal ions by phytic acid, the bio-availability of these ions from food gets limited in humans and other monogastric animals. Reduction of phytic acid content is hence, an important breeding goal in crop plants.

Many natural mutants with reduced phytate in different crops have been identified. However, reduction in phytic acid content has been associated with negative pleiotropic effects like decreased germination percentage and weight of seeds. Inositol phosphates play an important role in several cellular functions, including lipid metabolism. Impairment of the metabolic functions, otherwise carried out by inositol phosphates, in the phytic acid-related natural mutants could be a reason for their negative pleiotropic effects^6^. In this context, the challenge is to reduce the phytic acid levels without introducing substantial negative pleiotropic effects. Therefore, it is important to choose the best gene target(s) to accomplish the desired outcome, which is reduced phytic acid content with minimal off-target effects. Raboy has summarized the pathway of phytic acid synthesis, starting from glucose-6-phosphate and different targets have been indicated for reducing phytate^7^. Various natural mutants like soybean LR33; maize *lpa3-1*, *lpa2-2*, *lpa1-1*; *Arabidopsis* *atipk1*, *atipk2* etc. can be potentially targeted. The mutants in various crops, the responsible loci, amount of phytate reduction and other phenotypic effects associated with the mutant have been summarized in Sparvoli and Cominelli^6^. Three classes of maize mutants with reduced phytic acid content, referred to as *low phytic acid* or *lpa*, are known. Raboy *et al*. have described the origin and phenotype of seed phytic acid mutants *lpa1* and *lpa2* in maize^8^. Shi *et al*. characterized *lpa3* mutants of maize^9^. In terms of phytic acid biochemistry, *lpa3* is the most upstream and encodes a *myo*-inositol kinase, which phosphorylates *myo*-inositol to inositol monophosphates. *lpa2* encodes Inositol Phosphate Kinase 1 (IPK1), which phosphorylates inositol-5-phosphate (IP_5_) to inositol-6-phosphate (IP_6_) or phytic acid. *lpa1* is the most downstream of all and encodes an ABC (ATP binding cassette) - transporter protein that packages phytic acid into various vacuoles. In many natural mutants, the expression of the encoded protein is switched off. Proteins are usually involved in interactions with many other proteins. Null expression or silencing of a protein results in disruption of protein-protein interactions and signaling or downstream metabolic networks associated with such protein-protein interactions. Disruption of protein-protein interaction networks comprises a potential reason for the diverse negative pleiotropic effects linked with natural or engineered mutants. Raboy has enlisted four potential target areas, which include (a) inhibition of the synthesis of *myo*-inositol and inositol-3-phosphates, (b) inhibition of synthesis of inositol-6-phosphate (phytic acid), (c) inhibition of transport and storage of phytic acid in the cell, and (d) expression of phytase encoding transgenes^10^. Given the public aversion to genetically modified foods in certain countries, the last approach is limited in practical value, hence the first three may be considered for a broader applicability. Genome editing gives an opportunity to modify specific gene(s) to achieve desirable effect^11^. In this regard, it is essential to prioritize target gene(s), for achieving precise engineering of physiological processes with minimal negative pleiotropic effects associated with changes in a particular gene sequence(s). One possible strategy to avoid off-target effects is to mutate a protein through gene editing to result in a variant that has reduced or null enzymatic activity. The edited protein would thus block the concerned metabolic reaction. However, the protein would still be fully expressing to fulfill its function of interacting with other proteins. Targeting the upstream reactions of a metabolic pathway would result in a snow-ball effect, influencing all the other downstream processes. Hence, inhibition of *myo*-inositol and inositol-3-phosphates would disrupt not only the inositol metabolism, but all other allied processes associated with it. Inositol phosphates are involved in a range of cellular functions like membrane transport, cell division, cytodifferentiation, regulatory role in signal transduction (lipid signalling) etc^12^. On the other hand, blocking the transport of phytic acid in cellular compartments has been described as a viable strategy. Shi *et al*. silenced embryo-specific expression of an ABC transporter protein that transports phytic acid from cytoplasm to vacuole^13^. The authors report that while the null *lpa1* mutant has attenuated seed germination, the *lpa1-1* point mutant displays normal germination percentage. However, the seed weight of point mutant is lesser than normal maize. Landoni *et al*. have demonstrated changes in physical properties of *lpa1* maize, including modifications in density, starch properties, fiber content etc., which has implications for the practical utility of *lpa1* maize^14^. The available evidence shows that phytic acid can be packaged into cell organelles via multiple transporter proteins of the ABC Multiple Drug Resistance associated Protein (MRP) type. In *Arabidopsis*, the phytic acid is known to be packaged in at least three compartments: protein storage vacuole of embryo, endoplasmic reticulum and vacuolar compartments of chalazal endosperm^15^. The storage in last two compartments is transient. Due to multiple transport compartments, silencing of one transporter protein in one compartment may not be a viable strategy. Further, the accumulation of phytic acid in cytoplasm has the potential to lead to toxicity, as phytic acid at defined concentrations has been demonstrated to possess cytotoxicity in human cell lines^16^. Either or combination of the above phenomenon may be responsible for the decreased seed weight of *lpa1* mutants.

**Figure 1:**
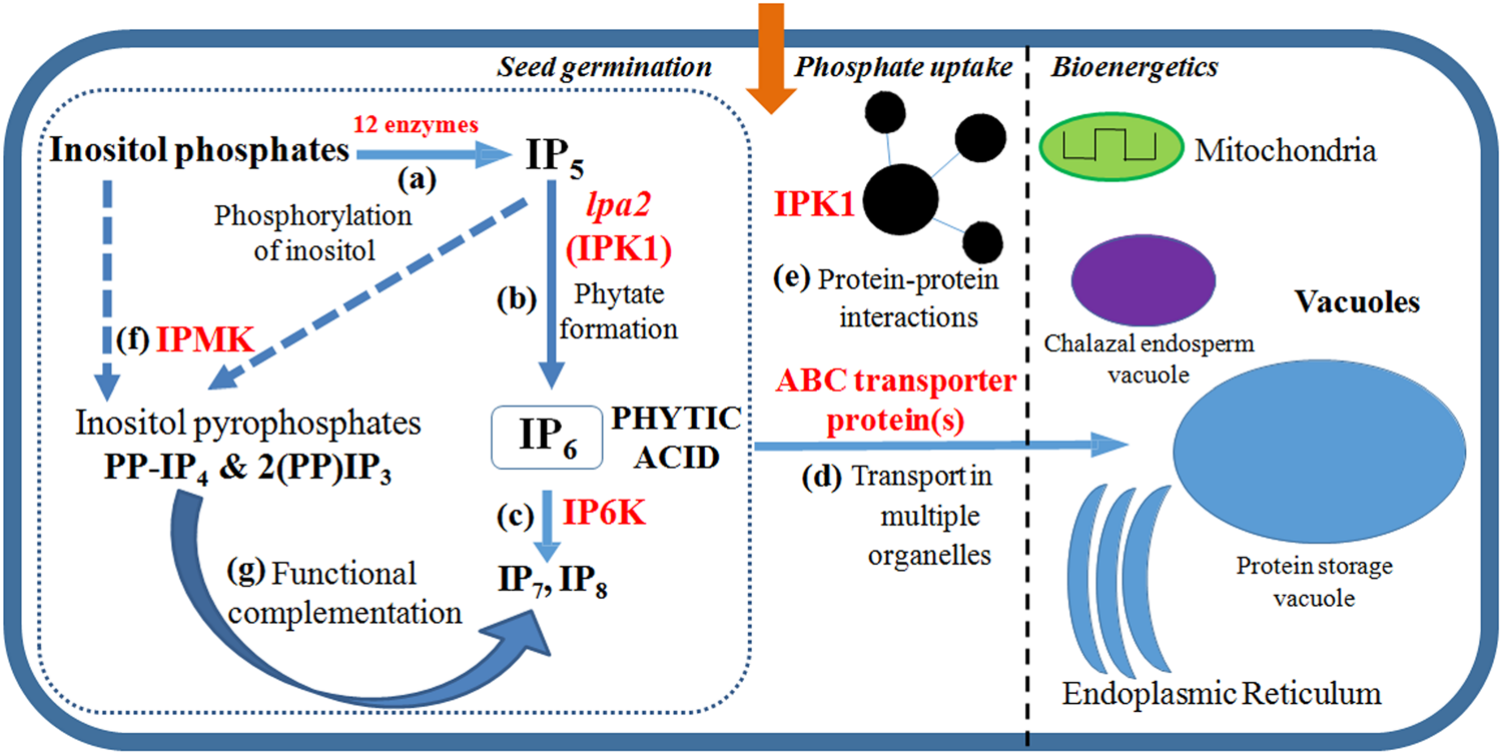
A model of key components of inositol phosphate pathway involving IPK1. (**a**) *myo*-Inositol is converted to inositol phosphates, including IP_5_ via 12 enzymes. **(b)** IPK1 then phosphorylates IP_5_ to IP_6_ or phytic acid. **(c)** IP6K phosphorylates phytic acid to higher forms like IP_7_ and IP_8_. **(d)** Phytic acid is transported to different organelles via ABC transporter proteins into storage vacuole, chalazal endosperm vacuole and endoplasmic reticulum. **(e)** IPK1 is involved in protein-protein interactions with other proteins, which are involved in seed germination, phosphate uptake and bioenergetics. **(f)** Inositol phosphate multi-kinase ( IPMK) converts IP_5_ and other inositol phosphates to inositol pyrophosphates PP-IP_4_ and 2(PP)-IP_3_. **(g)** Inositol pyrophosphates PP-IP_4_ and 2(PP)-IP_3_ are capable of mediating functions carried out by IP_7_ and IP_8_, which would not be formed in the event of absence of IP_6_. IPK1 itself, rather than a single transporter protein or an upstream enzyme, appears to be the most promising target for low-phytate maize.

Being the most downstream enzyme in the metabolic pathway of phytic acid formation, Inositol Phosphate Kinase 1 or IPK1 is a potential gene target for achieving reduction in phytate content. IPK1 catalyzes the phosphorylation of inositol phosphates to higher phosphorylated forms like IP_6_ (Phytic acid). It is also involved in protein-protein interactions with other proteins. IPK1 can thus be mutated, such that the phosphorylation of different inositol phosphates does not take place, but the protein remains intact for protein-protein interactions to take place. Figure 1 shows the current understanding of phtyic acid formation and transport in cell. Studies have shown that apart from inositol phosphates, inositol pyrophosphates also play an important role in cellular metabolism^17,18^. Phytic acid is further converted to higher phosphorylated forms by Inositol hexakisphosphate kinase (IP6K) enzyme. This leads to the formation of inositol pyrophosphates IP_7_ (PP-IP_5_) and IP_8_(2(PP-IP_4_)^19^. The available literature suggests that the function of IP_7_ and IP_8_ can be carried out by inositol pyrophosphates PP-IP_4_ and 2(PP)IP_3_^19^. Saiardi *et al*. have shown that a null mutant of IPK1 (*ipk1Δ*) in yeast results in accumulation of pyrophosphates PP-IP_4_ and 2(PP)IP_3_ besides IP_5_, yet resembles wild-type cells in morphology^19^. Abnormal vesicular morphology in case of null mutations of other inositol phosphate kinases is attributed to the loss of inositol pyrophosphates, which does not happen in case of *ipk1Δ*, thus explaining its similar morphology to wild type cells. Hence, loss of phytic acid would not impair the function mediated by IP_7_ and IP_8_, since PP-IP_4_ and 2(PP)IP_3_ impart functional redundancy by complementation [Fig. 1(f,g)]. In view of the above and multiple transport routes of phytic acid along with protein-protein interactions of IPK1, it appears to be the critical kinase which can be targeted without introducing multiple negative plieotropic effects, as observed with other mutants. The IPK1 enzyme can be minimally altered by disrupting its catalytically active site, so that the protein is fully expressing, but unable to convert IP_5_ to phytic acid. The desired minimal alteration in IPK1 would only affect phytic acid formation, leaving the upstream and downstream process, as well as the protein-protein interactions intact.

**Figure 2:**
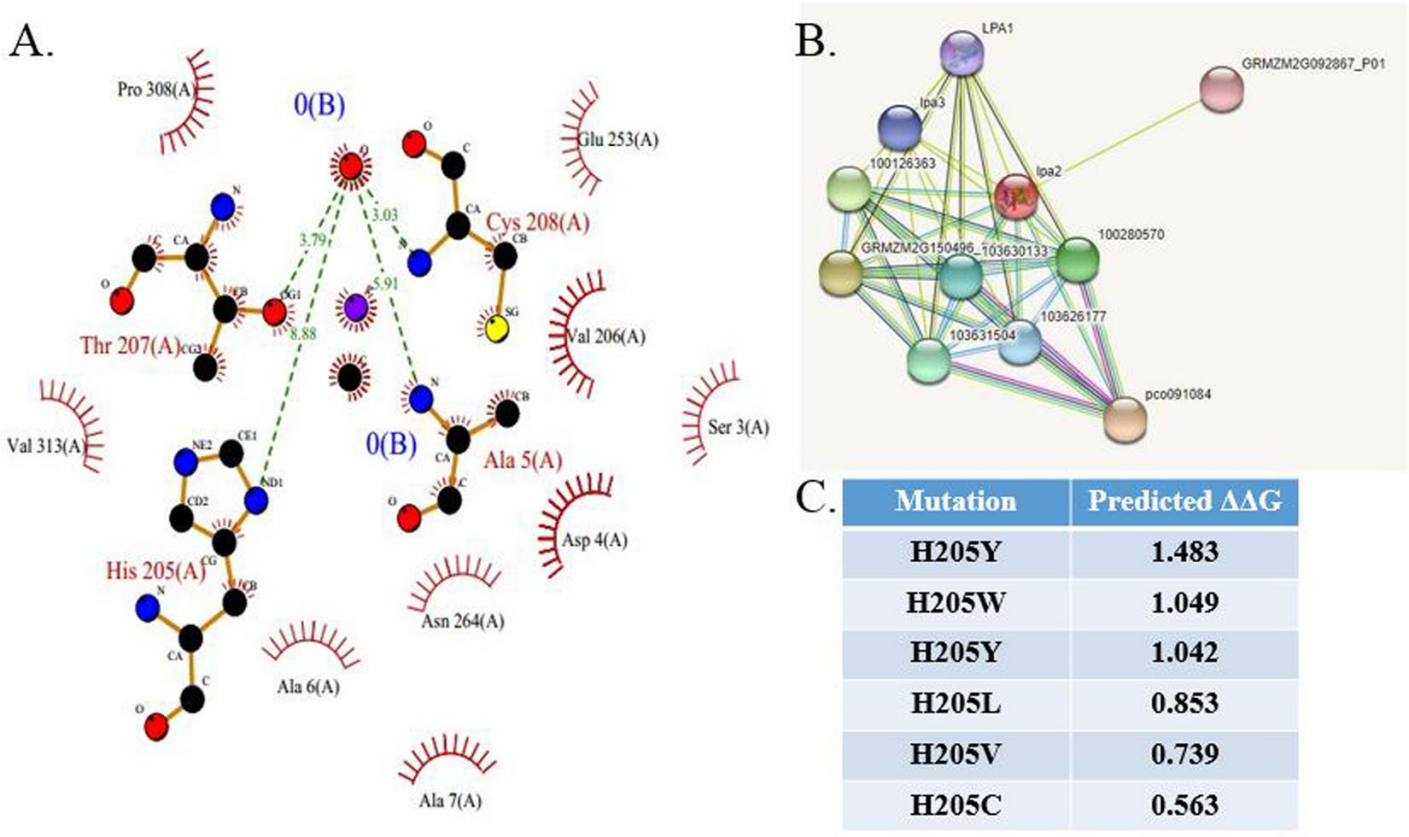
Computational model of IPK1 with its substrate, its interacting proteins and identification of key mutations for catalysis inhibition. (**A**) Residue of IPK1 that interact with substrate IP_5_. (**B**) Protein-protein interactions of IPK1 indicating associated proteins, revealed by databases and textmining. (**C**) Mutations that destabilize protein at 205 position and have the potential to hinder catalytic activity.

In order to evaluate the prospects of minimally altering *Zea mays* IPK1, a computational model of the protein was made using PSI-BLAST (Position-Specific Iterative Basic Local Alignment Search Tool) based structure prediction^21^. The structure was refined by side-chain repacking^22^. The refined structure contains 13 α-helices and 15 β-sheets, with 92.4% residues in Rama-favoured region and no poor rotamers. The Class (C), Architecture (A), Topology (T), superfamily (H) analysis^23,24^ of the modeled *Zea mays* IPK1 structure showed it to contain the structure typically found in inositol phosphate kinases. The substrate inositol pentkisphosphate (IP_5_) was docked to the IPK1 model using a rigid docking algorithm^26,27^, which was further refined^28,29^. Both IP_5_ and cofactor Adenosine triphosphate (ATP) bind in a cleft formed by four β-sheets from residues 195–202, 205–212, 278–286, 292–299 and two α-helices 260–273 & 310–326. Analysis of the docked structure using PDBsum web server ^30,31^ showed Alanine 5, Histidine 205, Threonine 207 and Cysteine 208 to be closely interacting with IP_5_ (Fig. 2A). The proteins that interact with IPK1 via protein-protein interactions include acid phosphatase, inositol-pentakisphosphate 2-kinase, inositol polyphosphate multikinase, succinate-CoA ligase, inositol 3-kinase, ABC MRP4 transporter and a metal ion binding protein (Fig. 2B). Mutation of the IPK1 protein at key amino acids that result in destabilization of the protein in its active site or hinder interactions with substrate or cofactor will result in a protein, functional for protein-protein interactions but non-functional for phytic acid formation. In the present case, Histidine 205 is implicated to be important for protein stability. Various mutations at His205 position have the potential to destabilize the protein, thereby hindering its function of phosphorylation (Fig. 2C). Similarly, alanine mutants of other interacting residues have the potential to inhibit phosphorylation by IPK1.

Cowieson *et al*. have postulated a term ‘phytate-free nutrition’ to emphasize the importance of reducing the content of phytic acid in feed^20^. The authors mention the fact that prominence is being given to formulation of phytate-free diets rather than accommodating phytase enzyme in the diet, as majority of phytic acid is digested in ruminants, but recalcitrant phytic acid does not get digested. If phytic acid content is reduced in the first place, mineral bioavailability would be enhanced. At the same time, efforts must be directed to minimize any possibilities for off-target effects of phytate reduction on plants. The above study is a step towards prioritization of target genes for dephytinization of maize to enhance its nutritional value.

## Methods

### Generation of protein model

The DNA sequence of *lpa2* encoding for inositol phosphate kinase 1 was taken from National Center of Biotechnology Information. It was modelled through Bioserf (available on PSI-PRED webserver)^21^. The obtained model was refined through GalaxyRefine program^22^. The modelled structure was analysed for class, architecture, topology, superfamily through CATH/Gene3Dv4.2 program^23,24^.

### Docking of substrate and cofactor with protein

The substrate inositol pentakisphosphate (IP_5_) and cofactor Adenosine triphosphate (ATP) were downloaded from ZINC database^25^. The substrate and cofactor were docked on to the modelled protein using Patchdock algorithm^26,27^ and refined using Firedock webserver^28,29^. The interacting residues were identified using PDBsum^30,31^.

### Protein interactions and prediction of stability at key residues

The protein-protein interactions of IPK1 were obtained using STRING database^32^. The stability of protein with mutation at key residues was determined using Site Directed Mutator program^33^.
